# Photonic angular momentum: progress and perspectives

**DOI:** 10.1515/nanoph-2022-0035

**Published:** 2022-02-18

**Authors:** Andrew Forbes, Siddharth Ramachandran, Qiwen Zhan

**Affiliations:** School of Physics, University of the Witwatersrand, Private Bag 3, Wits 2050, South Africa; Boston University, 8 Saint Mary’s St., Boston, MA 02215, USA; School of Optical-Electrical and Computer Engineering, University of Shanghai for Science and Technology, Shanghai 200093, China

## 1 Introductory remarks

Although the existence of photonic angular momentum is imbedded in Maxwell’s equations, the full picture of its intricate connections was slow to emerge. Early work on polarization and energy flow were connected by Poynting [1] and used to predict the mechanical potential of spin angular momentum (SAM) from circularly polarized light, later confirmed by Beth [2]. Although photonic orbital angular momentum was very well known since the early days of quantum mechanics, creating photons with orbital angular momentum (OAM) was restricted to unlikely quadrupole transitions in atoms, and remained the realm of textbook atomic physics until only 30 years ago [3]. This seminal work revealed the importance of the spatial structure of light, particularly its helical phase, and its connection to OAM. The explosion of activity that followed was fueled by the ease by which OAM light could be created, bringing photonic angular momentum tools into optical laboratories.

Almost in parallel, vectorial optical fields with structured polarization, particularly those with cylindrical polarization symmetry and polarization singularity received lots of attention mainly fueled by their focusing properties [4], and their intrinsic links to SAM and OAM have been investigated through spin-orbital conversion, coupling and interactions. The large emergent community has steadily uncovered further connections between the many degrees of freedom in structured light [5], spatial and temporal, and its angular momentum, including spin–orbit coupling [6, 7], geometric representations of photonic angular momentum states [8], transverse angular momentum [9], spatiotemporal vortices (STOV) [10] and exotic photonic wheels [11]. Despite the progress [12, 13], there remains much to do, with open challenges and opportunities.

In celebration of 30 years since the seminal work on OAM [3], in this special issue we collect authoritative reports on the present state-of-the-art in the science and applications of Photonic Angular Momentum.

## 2 Special issue synopsis

The special issue includes technical and historical reviews by leaders in the field, touching on the nature of twisted light and its potential as an optical analogy to other physical effects [14], and the extensive use of photonic angular momentum in optical communication [15], including free-space, fiber and underwater. Exotic beams based on photonic angular momentum are reported, including accelerating vectorial beams [16], synthesis of partially coherent beam with controllable twist phase [17], by-design 3D spin and OAM densities [18] and polarization transformations in free-space propagation by metasurface induced frozen waves [19]. Interesting phase and polarization singularity connections continue to be studied, such as angular momentum redirection phase of vector beams in a nonplanar geometry [20]. Developments in the very recently discovered transverse OAM contained within STOV are also reported, including lateral shift and time delay of STOV upon reflection and refraction [21] and tilting the orientation of OAM embedded within STOV using astigmatic mode converter [22]. The toolkit too has developed, including linear tools using digital micro-mirrors [23] as well as a nonlinear toolkit for the creation of quantum states by spontaneous parametric down-conversion [24] and classical angular momentum state conversion in up-conversion [25]. Deep-learning based technique is used to recognize multi-singularity within structured light [26]. Strong efforts in miniaturization of devices exploiting photonic angular momentum are evidenced by reports on the use of reconfigurable terahertz metasurface for structured light [27], metasurface decorated with optical crystal for nonlinear wavefront engineering [28], nanostructured silica optics for modal vortex shaping [29], on-chip optical polarimeter for spin separation [30], photonic integrated chip OAM multiplexer [31] and nanofabricated lithium niobate fork grating [32]. Drives for ultrafast and high-power sources are demonstrated with a high-power thin-disk laser for cylindrical vector modes emission [33] and a megawatt femtosecond optical vortex mode fiber based Mamyshev oscillator [34]. Finally, researchers continue to push the applications of photonic angular momentum in various areas, including fabrication of hexagonal close-packed ring-shaped structures [35], angular velocity measurement based on OAM [36], and communications that are either fiber based [37] or through free space [38].

## 3 Progress and perspectives

The special issue content was limited to just one by-invitation contribution per leading group, and consequently the scope is necessarily limited. An exciting trend in photonic angular momentum not extensively covered is the creation such exotic states of light directly at the source, from lasers. Controlling the polarization state from a laser is routine and the subject of laser textbooks, but other forms of angular momentum control have been problematic. Structured light from lasers [39] has enjoyed a resurgence only lately in the context of OAM [40, 41]. Although many attempts were made in the early days of the laser to create Laguerre–Gaussian modes [42], this was not in the context of photonic angular momentum. The first OAM reported “at the source” was from a bulk gas laser using a spiral mirror [43], exploiting the propagation phase of light. Amplitude filtering has seen several approaches, but control of the handedness of the OAM required helicity induced losses to be engineered [44]. Geometric phase control was introduced much more recently [45], with intra-cavity q-plates [46] allowing scalar OAM helicity control as well as vectorial combinations to be created from the same device. Advances in metasurfaces has seen the emergence of J-plates for total angular momentum control through a combination of geometric phase and spin controlled propagation phase [47]. Inside a laser, such devices allow the creation of super-chiral light with high OAM (shown up to 100) and arbitrary values (100 and 10 for example) coupled to linear polarization states [48]. It is exciting to see what further developments these nano-structured optics will bulk bring to photonic angular momentum lasers. Rather than using intra-cavity exotic optical elements, the geometry of the laser can be used to create tailored photonic angular momentum, both in large solid-state lasers [49] and in on-chip devices [50].

The challenge is to push these devices in power, versatility and robustness, so that they can move from research projects to practical devices. Versatility and robustness have shown progress of late, with topological photonics based solutions ushering in new approaches for the control of OAM by the SAM of the pump, for on-demand helicity control [51]. Compact fiber lasers for scalar and vectorial OAM combinations have too been demonstrated allowing tuneability in both wavelength and OAM [52]. But most of these lasers are still at very low powers, with the exception of the gas, disk and fiber laser systems that have produced cylindrical vector modes (radially and azimuthally polarized), reaching several kilowatts of average power and near 100 GW in peak power, an example of which features in this very issue [33]. A promising prospect to amplify the low power states, which has already seen developments in thin disk, fibers and bulk crystals, with recent state of the art mimicking amplification of ultrafast lasers to demonstrate vectorial light parametric amplification in a polarization insensitive manner, reaching 1000-fold amplification factors [53].

On the other extreme are quantum forms of photonic angular momentum. SAM has enjoyed a rich history in quantum optics, but only recently has other forms of angular momentum come to the fore. Seminal work dates back to OAM entanglement 20 years ago [54], with hybrid spin–orbit states introduced far more recently [55]. Using general structured photonic angular momentum as a basis for expressing entanglement has seen significant progress in the science and applications of such quantum states of light [56]. Despite this, while SAM has seen heroic experiments involving 100s of entangled photons, and communication across 1200 km, OAM and hybrid states languish at few photon entanglement experiments [57], 10 dimensions in teleportation [58] and distances in the order of 300 m in free space [59] and low kms in optical fiber [60]. The challenge is to improve our toolbox for the creation and detection of such quantum states, as well as harness these new degrees of freedom for quantum information processing and communication. An example of such steps is shown in this issue using the work horse of entanglement, spontaneous parametric down-conversion [24].

Emerging topics that involve structured light and photonic angular momentum continues to draw significant amount of attention. In the past, structured light and the associated photonic angular momentum studies have been mostly associated with light modulations in the spatial domain. Arbitrarily vectorial optical fields that contain complex photonic angular momentum distributions and networks can be designed and generated. On another front, pulse shaping techniques have been developed to produce specific pulse waveforms in the temporal domain to meet the needs of various applications. However, the spatial and temporal structuring of light are mostly developed in parallel, even though pulsed structured light can be produced by combining these two to form wavepackets that is mathematically separable in space and time.

Recently there has been a dramatic increase in research interests in spatiotemporal coupled optical fields, a more general type and mathematically nonseparable spatiotemporal pulses. These fields feature unique photonic properties that are previously unavailable from conventional optical fields. Particularly, pulse shaper method for the generation of spatiotemporally coupled optical field [61] has seen great success and received tremendous attention. Spatiotemporal optical vortex (STOV) that carries transverse OAM perpendicular to the light propagation direction was theoretically predicted earlier [62], whose controllable generation using a pulse shaper configuration was only reported very recently [10]. This development quickly drew lots of attention and several important advances have been reported since then, including conservation of transverse OAM during propagation [63, 64], conservation of transverse OAM through nonlinear optical processes [65, 66], partially coherent STOV [67], and generation of STOV using transmission nodal line [68], OAM with controllable orientation [69], Bessel STOV [70], ultrafast modulation of transverse OAM [71], and so on. Related progresses are also reported in this special issue, including lateral shift and time delay of STOV upon reflection and refraction [21] and tilting the orientation of OAM embedded within STOV using astigmatic mode converter [22]. These latest developments of STOV and transverse OAM firmly put the OAM on the equal footing as the SAM and the last piece of the puzzle for the photonic angular momentum is finally in place.

Despite these great successes, it may be just the tip of an iceberg and much more needs to be studied, understood and developed. The pulse shaper technique is based on the use of spatial light modulator at the spatial-frequency domain of the optical field. Recently, this method has been extended to generate much more complicated STOV lattices with chirped pulses [72]. In addition, the combination of the spatiotemporal modulation of light with the already mature spatial manipulation of light offers unprecedented degrees of freedom in optical field modulation, enabling the generation of a variety of novel optical wavepackets such as cylindrical vector STOV [73], photonic toroidal vortex ring [74], optical toroidal [75], and so on.

A majority of the investigations with light’s angular momentum since the pioneering demonstrations of Allen and co-workers [3] have concerned themselves with bulk media or free-space. In this regime, OAM and SAM are often separable quantities, their linear combinations forming mutually unbiased bases. However, given the well-understood phenomena of Imbert–Federov shifts [76], light incident at interfaces accumulates phases dependent on its OAM and SAM state. Such, so-called spin–orbit interactions (SOI) have been observed at interfaces, during high numerical-aperture focusing [77], or in light–matter interaction at the nanoscale [78, 79]. A medium where this effect is readily observed is an optical fiber. The refractive index inhomogeneity of an optical fiber that acts as an attractive potential interacts with light to yield the classical L⋅S coupling reminiscent of the splitting of energy levels of electrons in confined potential wells [80]. The result is that OAM and SAM states that are degenerate in bulk media and in free-space acquire a SOI-induced splitting of their propagation constant. Carrying the analogy of photons in a (circularly-symmetric) waveguide with electrons in a confining potential further, one can also see that, just as the effective mass of an electron increases with it OAM magnitude, the group-velocity dispersion of light is also strongly dependent on, and increases with, its topological charge. Hence, optical fiber propagation of light carrying angular momentum provides for an additional tool to tailor its phase as well as group-velocity [81]. Moreover, SOI also enables engineered chirality in an otherwise isotropic medium, yielding a new platform to control light’s propagation-dependent polarization state [82]. Since control of these parameters of light is of paramount importance in the field of nonlinear optics, fiber-based control of the OAM and SAM content can profoundly enhance nonlinear interactions and also yield new modal selection rules. Examples include the ability to create a large ensemble of photon pairs, the ability to scale the power of OAM beams to kW peak power levels at nontraditional wavelengths [83], novel phase-matching criteria for Raman scattering where light’s OAM can control the bandwidth [84], spectral shape and strength of Raman interactions, and phase conjugation via simulated Brillouin scattering [85].

For almost 20 years since investigations into light’s OAM commenced, following the work by Allen and co-workers, the aforementioned SOI and OAM dependent propagation constant was actually the source of a debilitating problem for achieving fiber propagation of OAM states. The inherently large density of states in an optical fiber combined with the fact that propagation over any reasonable lengths of a fiber results in bend and twist perturbations [86], meant that OAM states would uncontrollably mix, yielding speckle patterns that did not maintain OAM or SAM in any desired state. The solution to this problem was found in 2009, with ring-core fibers whose index profile mirrors the intensity distribution of OAM modes [87]. This design, instead of minimizing the debilitating effect of SOI, exacerbated it so that phase and group-velocity differences between different linear combinations of OAM and SAM states were maximized. This, in turn, dramatically reduces coupling between different eigenmodes of an optical fiber. Since that demonstration, ring-core index profiles, featuring nanobores [88], inverse gradient index profiles [89], air-cores [90] and other similar designs have become the mainstay of optical fiber design to enable OAM and SAM mode propagation. Gain-doped versions of these fibers have yielded optical amplification of OAM modes [91, 92], and have also been used for developing high-power lasers featuring emission in an OAM state [93]. Since such designs yield a large ensemble of independent channels in an optical fiber, they have particularly found utility in the transport of classical [94], [95], [96], [97] and quantum information [60] over lengths exceeding kilometers.

It is never wise to predict what would come out from a nascent research field such as spatiotemporally sculptured optical field. However, we may be able make some intelligent guesses of the future development of this emerging field by drawing parallels with the development of spatially structured light. For example, new generation methods that utilize metasurfaces or other nanophotonic structures are highly desirable in order to miniaturize the generation system and incorporate modulation of multiple parameters into the system. With multiple-parameters modulated spatiotemporal fields, new characterization method will be needed. Consequently, a more comprehensive set of toolboxes for the generation and characterization of these complex fields would have to be developed. With such a toolbox available, many other spatiotemporal optical solutions that are highly interesting but was not possible to generate currently, such as those localized waves [98], may become feasible. Another important future direction for spatiotemporally sculptured optical fields is to study novel light–matter interaction phenomena with the generated optical fields. Turning our attention to fiber propagation of OAM and SAM modes, current advances have already yielded new nonlinear optical selection rules, more efficient and selective nonlinear optics, as well as the means of developing high-power sources possessing photonic angular momentum. It is conceivable that these new “knobs” with which to tailor nonlinear optics would play an important role in source generation, in the quantum as well as classical regimes. An important question to address is whether there is a limit in the dimensionality – i.e., the number of stable PAM modes – that one can achieve in optical fibers, because this directly impacts the multitude of applications across nonlinear optics, sensing, and classical and quantum networks where OAM modes in fiber has already had considerable impact. The largest ensemble of mode-mixing-free OAM modes over which km-length pulse propagation has been demonstrated [96] is 12, as of this writing. Enhanced SOI effects, where not only the propagation constant, but the fields are also perturbed, has shown that as many as 24 modes may be transmitted stably over 10 m of fiber [7], though it is not clear if this conceptual framework is scalable in mode count or fiber length. Continuing work in the field will potentially find new waveguiding mechanisms, or optimization of fiber index profiles, to substantially increase the mode count of stable OAM and SAM modes in optical fibers. And finally, how to transfer the spatiotemporally coupled optical field technique to super-intense and ultrafast high power laser deserves special attention as it may hold the key to unlock the full potential of these large laser facilities. With the unprecedented level of control of the spatiotemporal degree of freedoms of light, spatiotemporally sculptured optical fields will significantly enrich the photonics arsenal for scientists in broad research fields ranging from quantum optics, nanophotonics, spin-photonics and spintronics, optical information transmission and processing, optical spectroscopy, laser driven particle acceleration, and much more beyond.
